# Œsophagotomy1Read before the Keystone Veterinary Medical Association.

**Published:** 1899-02

**Authors:** W. H. Ridge

**Affiliations:** Trevose, Pa.


### ŒSOPHAGOTOMY.[1]

By W. H. Ridge, V.M.D.,
TREVOSE, PA.

This operation is performed by many, perhaps, yet the results are seldom recorded. It is easy to perform, yet many complications often follow it.

The indications for operation are frequently most urgent; often the choice rests between operating and leaving the animal to die. Then we should not hesitate to operate.

The indications for this operation are in any case where the animal is unable to swallow, as in tetanus, spinal meningitis, severe pharyngitis, also in choke or wounds of the œsophagus.

[1] Read before the Keystone Veterinary Medical Association.

The œsophagus lies between the trachea and the longus colli; in the middle cervical becomes external to trachea, toward the left side.

In performing the operation we need not cast the animal. If a horse, a twitch will give us all the restraint necessary in most cases. In operating to relieve starvation in strangles and tetanus we make an incision in the middle cervical region, just below the jugular groove, passing through the skin, panniculus and connective tissue, directing our course posterior to the trachea until we find the œsophagus. We then insert a trocar, feeding the animal through the canula. We suture the canula in the wound, leaving it in the œsophagus until the animal is able to swallow. This operation will often give the animal relief, with very little added danger.

Our next operation on the œsophagus is for choke. This is a much more serious operation. I have performed it several times, and think it more prudent to operate than use too much force with the probang. While Professor Müller advises us to make our incisions below the jugular vein, I have made mine above. I think it more difficult in finding the œsophagus in the lower operation. Also, he says, "the œsophagus should be separated as little as possible from its surroundings." While this is literally true, it may lead us to difficulties, for we will want the œsophagus outside the wound, and if we make our incision through the œsophagus onto the substance that causes the choke, which, by the way, causes the œsophagus to protrude, we will find it very difficult to suture, and most likely make a failure.

The most simple operation for the relief of choke is to enucleate the œsophagus, then make a small incision below the object, the incision being only large enough to pass a blunt-pointed knife; then pass the blunt-pointed knife in and to the opposite side of the object, cutting it in half, which mostly will give relief, the small size of the incision giving us but little trouble.

My method of operation where the object is too solid to cut, as above described, is to enucleate the œsophagus, turn the œsophagus so that we can make an incision on the inner side—that is, the part lying toward the longus colli, of course, making the incision lengthwise of the gullet. After suturing very closely, thoroughly wash with disinfectant. After drying, powder with boric acid and iodoform; suture externally; give no solid food for several days.

CASE I.—Heifer choking on turnip. After trying to push it down with the probang, with free use of oil, not succeeding, and the animal suffering badly, I performed œsophagotomy, cutting down to the œsophagus. Making a small hole in the œsophagus, I passed a probe-pointed bistoury and cut the turnip in two, but in doing so, the animal being restless, in a standing position, and having only a lantern to furnish the light, I cut the opening about one inch long. It being my first operation of this kind, I did not enucleate the œsophagus. I could not suture it, but left an open wound. The animal was fed sloppy food for about one week, when there was but little discharge; then the animal aborted, lost flesh and strength rapidly, and died in two weeks from the time of operation.

CASE II. shows us that there are cases that are left to die when an operation may save them. The subject was a cow choked November 29th of last year. The owner had tried to relieve her by passing a carriage-whip.

On arrival I found her very tympanitic; punctured rumen with trocar. On passing probang found that the choke was about three inches posterior to pharynx. On removing the probang I found the end covered with blood. I then knew there was a laceration if not a complete rupture of the œsophagus. The passing of the probang gave the animal such pain that it was impossible to move it in that manner. I offered to operate, which met the views of the owner. The animal was cast on the right side, a longitudinal incision was made above the jugular gutter, passing back of the carotid. I enucleated the œsophagus, when I found a rupture on the inner aspect of the tube, with a corner of a half of a turnip protruding. I increased the length of incision to two inches, cut the turnip in half, and took it out. I then washed out the wound with creolin solution, then sutured closely; again washed it out, and dried with a sponge, then packed with boric acid with a little iodoform; then sutured the skin. The animal was fed bran-mash, boiled oats, and soft feed.

On December 2d there had been no discharge or swelling that one would notice in passing through the stable. The sutures in the œsophagus were of silk, and never gave any trouble. The wound healed without discharge, and the cow never missed a feed. On February 13th calved without trouble, and has remained healthy since.

---

Dr. Charles E. Tomlinson, of Liberty, Pa., was a visitor to the JOURNAL office in January.

